# Supplementary material for: Identification of photoperiod-induced specific miRNAs in the adrenal glands of Sunite sheep (Ovis aries)
Source: Front Vet Sci. 2022 Jul 22;9:888207. doi: 10.3389/fvets.2022.888207 (PMC9354845; doi:10.3389/fvets.2022.888207)
Supplement: Supplementary file 1 [file Data_Sheet_1.ZIP › Supplementary file/miRDeep2 file.zip!/miRDeep2 file/Know_Structure/LP42A3/oar-mir-16b.pdf]

miRBase precursor : oar-mir-16b
Total read count : 37830
oar-miR-16b read count : 37771
remaining reads : 59

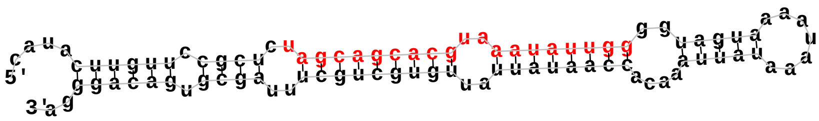

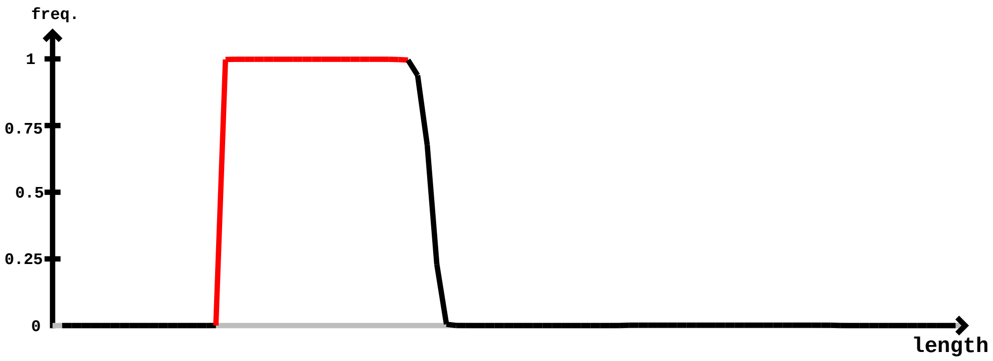

oar-miR-16b

5'- cauacuuguuccgcucuagcagcacguaaauauugggguaguaaaauaaauauuaaacaccaauauuauugugcugcuuuagcgugacaggga -3'	exp		
....((((((.((((..(((((((((..((((((((..(((((.......)))))....))))))))..)))))))))..)))).))))))..	reads	mm	sample
...............cuagcagcacguaaauauugggg...	3	0	seq
...............cuagcagcacguaaauauuggggu..	4	0	seq
................uagcagcacguaaauau..	1	0	seq
................uagcagcacguaaauauu...	41	0	seq
................uagcagcacguaaauauug..	71	0	seq
................uagcagcacguaaauauugg...	2166	0	seq
................uagcagcacguaaauauuggg..	544	0	seq
................uagcagcacguaaauauuggA..	9336	1	seq
................uagcagcacguaaauauuggAg...	15494	1	seq
................uagcagcacguaaauauugggg...	1423	0	seq
................uagcagcacguaaauauuggggu..	2893	0	seq
................uagcagcacguaaauauuggAgu..	5579	1	seq
................uagcagcacguaaauauuggggua...	177	0	seq
................uagcagcacguaaauauugggguag..	7	0	seq
................uagcagcacguaaauauugggguagu...	5	0	seq
.................agcagcacguaaauauugg...	1	0	seq
.................agcagcacguaaauauuggA..	6	1	seq
.................agcagcacguaaauauugggg...	1	0	seq
.................agcagcacguaaauauuggAg...	7	1	seq
.................agcagcacguaaauauuggAgu..	5	1	seq
.................agcagcacguaaauauuggggu..	8	0	seq
..................gcagcacguaaauauuggA..	1	1	seq
..................gcagcacguaaauauuggAg...	1	1	seq
..................gcagcacguaaauauuggggu..	1	0	seq
....................agcacguaaauauuggAg...	1	1	seq
....caccaauauuauugugcugc................	1	0	seq
....caccaauauuauugugcugcuu..............	1	0	seq
....caccaauauuauugugcugcuuu.............	1	0	seq
....caccaauauuauugugcugcuuua............	1	0	seq
....accaauauuauugugcugcu...............	2	0	seq
....accaauauuauugugcugcuu..............	4	0	seq
....accaauauuauugugcugcuuu.............	37	0	seq
....accaauauuauugugcugcuuua............	4	0	seq
....ccaauauuauugugcugcuuu.............	1	0	seq
....ccaauauuauugugcugcuuua............	1	0	seq

oar-miR-16b

```
cauacuuguuccgcucuagcagcacguaaauauuggguaguaaaauaaauauuaaacaccaauauuauugugcugcuuuagcgugacaggga
............................................................caauauuauugugcugcuuu.............    1    0    seq
```
